# Evaluation of Acute Pancreatitis in Patients Receiving Doxycycline Therapy: A Prospective Study

**DOI:** 10.7759/cureus.100808

**Published:** 2026-01-05

**Authors:** Aamir Nisar Gondal, Porus Ahmed, Faisal Akram, Arsallan Siddiqui, Hashmat Ullah Khan, Atika Hanif

**Affiliations:** 1 Medicine, Royal Blackburn Teaching Hospital, East Lancashire Hospitals NHS Trust, Blackburn, GBR; 2 Medicine, Mohi-ud-Din Teaching Hospital, Mirpur, PAK; 3 Family Medicine, SELECT Medical Clinics, Melbourne, AUS; 4 Medicine, SELECT Medical Clinics, Melbourne, AUS; 5 Medicine, Lady Reading Hopital MTI Peshawar, Peshawar, PAK; 6 Medicine, People's University of Medical and Health Sciences for Women, Nawabshah, PAK

**Keywords:** antibiotic toxicity, drug-induced pancreatitis, idiosyncratic reaction, pancreatic enzymes, pharmacovigilance

## Abstract

Background

Acute pancreatitis is an inflammatory disorder of the pancreas with diverse etiologies, among which drug-induced pancreatitis (DIP) represents a rare but clinically important subset. Doxycycline, a widely used tetracycline antibiotic, has been infrequently implicated as a causative agent in isolated case reports, leaving the true incidence and risk factors unclear.

Objective

This prospective study aimed to evaluate the incidence, clinical characteristics, and biochemical profile of acute pancreatitis in patients receiving doxycycline therapy and to identify potential associations between patient characteristics, duration of therapy, and disease occurrence.

Methods

This 24-month prospective observational study was conducted at Khyber Teaching Hospital, Peshawar, Pakistan. A total of 130 adult patients receiving doxycycline therapy were enrolled, of whom 124 completed follow-ups. Baseline pancreatic, hepatic, and renal enzyme profiles were assessed before therapy initiation, and patients were followed weekly for clinical symptoms and biochemical parameters. Acute pancreatitis was diagnosed according to the revised Atlanta criteria. Data were analyzed using IBM SPSS Statistics for Windows, Version 26 (Released 2018; IBM Corp., Armonk, New York, United States). Quantitative variables were expressed as mean ± SD, and categorical variables as n (%). Associations were evaluated using chi-square and t-tests, with p < 0.05 considered statistically significant.

Results

Among 124 participants, seven patients (5.6%) developed acute pancreatitis confirmed by biochemical and radiological findings. The mean latency to onset was 8.1 ± 2.3 days. The mean duration of therapy was significantly longer in affected patients (12.3 ± 2.1 vs. 10.6 ± 3.1 days; p = 0.03), while no significant associations were observed with age, gender, or BMI. All affected patients presented with classical symptoms, elevated serum amylase (422 ± 118 U/L) and lipase (698 ± 210 U/L), and recovered completely with conservative management. No mortality occurred.

Conclusion

Doxycycline-induced acute pancreatitis, though uncommon, is a clinically relevant adverse event with favorable outcomes upon early detection and drug withdrawal. Prolonged therapy beyond 10 days may increase risk, underscoring the need for clinical vigilance and patient monitoring during treatment.

## Introduction

Acute pancreatitis is an inflammatory disorder of the pancreas characterized by a sudden onset of epigastric pain radiating to the back, often accompanied by nausea and vomiting [1]. It remains one of the most frequent causes of hospital admissions for gastrointestinal emergencies worldwide. Although gallstones and chronic alcohol consumption account for approximately 70-80% of all cases, drug-induced pancreatitis (DIP) represents a small but clinically significant proportion, estimated between 0.1% and 2% of total cases [2]. Despite its rarity, the recognition of DIP is crucial, as timely identification and withdrawal of the offending drug can prevent recurrent or severe pancreatic injury [1].

Among the medications implicated in DIP, tetracycline antibiotics have been well documented, particularly tetracycline itself, which has been classified as a Class Ia agent due to strong evidence, including positive rechallenge cases [3]. Several other medications have also been associated with drug-induced acute pancreatitis, including azathioprine and 6-mercaptopurine, valproic acid, thiazide and loop diuretics, angiotensin-converting enzyme inhibitors, statins, corticosteroids, estrogens, antiretroviral agents, and certain chemotherapeutic drugs. However, doxycycline, a semisynthetic tetracycline derivative widely prescribed for respiratory tract infections, skin and soft tissue infections, and sexually transmitted infections, has only sporadically been linked to acute pancreatitis [4]. The currently available evidence largely comprises isolated case reports and a few small case series, as highlighted by several recent publications [4-9]. These case reports describe individual or limited cases of doxycycline-induced acute pancreatitis (DIAP), typically confirmed after excluding common etiologies such as gallstones, alcohol use, hypertriglyceridemia, and hypercalcemia. Most of these cases demonstrated mild to moderate disease, resolving after discontinuation of doxycycline and supportive management.

While these case-based observations have established a possible causal relationship between doxycycline use and acute pancreatitis, they are inherently limited by small sample sizes, anecdotal nature, lack of systematic follow-up, and the inability to estimate true incidence or assess risk modifiers such as dosage, duration of therapy, and patient demographics. The absence of prospective, systematically designed studies creates an important gap in understanding the actual frequency, risk factors, and clinical profile of this condition. Given the widespread clinical use of doxycycline, even a low incidence of pancreatitis can translate into significant morbidity if unrecognized. Therefore, the present prospective study was undertaken to systematically evaluate the incidence, clinical characteristics, and biochemical patterns of acute pancreatitis in patients undergoing doxycycline therapy at a tertiary care hospital in Pakistan.

## Materials and methods

This prospective observational study was conducted in the Department of Medicine, Lady Reading Hospital MTI, Peshawar, Pakistan, over a period of 24 months from January 2023 to December 2024. The study aimed to evaluate the occurrence and clinical characteristics of acute pancreatitis in patients receiving doxycycline therapy. Ethical approval was obtained from the Institutional Review Board (IRB) of Lady Reading Hospital MTI, Peshawar (Ref No: IRB/23/012, dated January 12, 2023). Written informed consent was obtained from all participants prior to inclusion in the study after explaining the study objectives, procedures, and potential risks.

The sample size was calculated using the OpenEpi sample size calculator (Dean AG, Sullivan KM, Soe MM. OpenEpi: Open Source Epidemiologic Statistics for Public Health), considering an expected prevalence of drug-induced acute pancreatitis of 5% [3], a confidence level of 95%, and a margin of error of 4%, resulting in a minimum required sample of 114 patients. To account for potential dropouts or incomplete data, a total of 130 participants were enrolled. Participant inclusion and exclusion processes were documented following the Strengthening the Reporting of Observational Studies in Epidemiology (STROBE) flow recommendations, ensuring clear reporting of eligible patients, excluded cases, reasons for exclusion, and final sample size used for analysis.

Patients were recruited using a non-probability consecutive sampling technique. All adult patients aged 18 years and above who were prescribed doxycycline for various medical conditions and consented to participate were considered eligible. Inclusion criteria comprised patients newly started on doxycycline therapy, with baseline normal pancreatic enzyme levels and no prior history of pancreatitis. Exclusion criteria included patients with a history of gallstones, chronic alcohol use, hypertriglyceridemia (triglyceride level >500 mg/dL), prior pancreatic disease, concurrent use of other known pancreatotoxic drugs (such as azathioprine or valproate), pregnancy, and patients with severe hepatic or renal dysfunction.

At baseline, demographic data (age, gender, BMI), clinical indication for doxycycline use, dosage, and duration of therapy were recorded. Indications for doxycycline therapy were restricted to clinically suspected or microbiologically confirmed bacterial infections, including respiratory tract infections, skin and soft tissue infections, and pelvic inflammatory disease; patients with suspected or confirmed viral infections were excluded at enrollment. Blood samples were collected prior to initiation of therapy to assess baseline serum amylase, lipase, liver function tests (alanine aminotransferase (ALT), aspartate aminotransferase (AST), alkaline phosphatase (ALP), and bilirubin), renal profile (urea and creatinine), and lipid profile. Baseline abdominal ultrasonography was performed in all participants to exclude pre-existing hepatobiliary disease, gallstones, ductal anomalies, or structural pancreatic abnormalities. Patients were followed up weekly during doxycycline therapy and for two weeks after therapy completion. Any symptoms suggestive of pancreatitis, such as abdominal pain, nausea, or vomiting, were recorded. In symptomatic patients, serum amylase and lipase were re-evaluated, and an abdominal ultrasonography or contrast-enhanced computed tomography (CT) scan was performed to confirm pancreatitis. Contrast-enhanced CT of the abdomen was selectively performed in patients where pancreatic anatomy was not adequately visualized on ultrasonography or where diagnostic uncertainty persisted.

Acute pancreatitis was diagnosed based on the revised Atlanta criteria (openly accessible and published under an open-access license, permitting unrestricted academic use) [10], which included the presence of characteristic abdominal pain, elevated serum amylase or lipase levels (≥3 times the upper limit of normal), and radiological findings consistent with pancreatitis. Infective causes of acute pancreatitis, including viral etiologies, were ruled out through detailed clinical history, absence of prodromal viral symptoms, and lack of laboratory or clinical evidence suggestive of viral infection at presentation. Potential sources of bias, such as misclassification bias, selection bias, and confounding, were minimized through prospective data collection, baseline enzyme screening, and standardized application of diagnostic and severity assessment tools, in line with STROBE recommendations.

Patients who developed acute pancreatitis were monitored for disease severity using the modified Glasgow-Imrie score (under standard copyright) [11] and were managed according to standard hospital protocols, including fluid resuscitation, analgesia, and nutritional support. Data on clinical course, hospital stay duration, complications (such as necrosis or pseudocyst formation), and outcomes were also recorded.

All biochemical investigations were performed in the hospital’s central diagnostic laboratory using standardized enzymatic colorimetric methods on an automated analyzer (Cobas c311, Roche Diagnostics, Mannheim, Germany). CT scans were interpreted by an experienced radiologist blinded to the clinical data.

Statistical analysis was performed using IBM SPSS Statistics for Windows, Version 26 (Released 2018; IBM Corp., Armonk, New York, United States). Quantitative variables, such as age, duration of therapy, and biochemical parameters, were expressed as mean ± standard deviation (SD), while categorical variables, such as gender, clinical manifestations, and incidence of pancreatitis, were presented as frequencies and percentages. The incidence rate of DIAP was calculated. Associations between the occurrence of pancreatitis and potential risk factors (age, gender, dosage, and therapy duration) were analyzed using chi-square or Fisher’s exact test for categorical variables and independent sample t-test for continuous variables. A p-value of less than 0.05 was considered statistically significant.

Confidentiality of all patient data was strictly maintained throughout the study, and no personal identifiers were disclosed. Participants developing acute pancreatitis received full medical management as per hospital protocol without any financial burden. This prospective observational study was conducted and reported in accordance with the STROBE guidelines. All essential components, including study design, setting, participant selection, variable definitions, data sources, bias control measures, statistical methodology, and handling of missing data, were explicitly defined and structured to meet STROBE reporting standards.

## Results

A total of 130 patients receiving doxycycline therapy were initially enrolled in this prospective study. Of these, 124 patients (95.4%) completed the entire follow-up period, while six patients (4.6%) were excluded due to loss to follow-up or incomplete data. The final analysis was therefore conducted on 124 participants. The mean age of the study population was 39.6 ± 12.8 years (range: 18-68 years). Among them, 74 (59.7%) were male, and 50 (40.3%) were female, with a male-to-female ratio of 1.48:1. The mean BMI was 25.3 ± 3.7 kg/m^2^. Doxycycline was most frequently prescribed for respiratory tract infections (60; 48.4%), followed by skin and soft tissue infections (34; 27.4%) and pelvic inflammatory disease (30; 24.2%). The mean duration of therapy was 10.7 ± 3.1 days, with a mean daily dose of 200 mg. Baseline demographic and clinical characteristics are summarized in Table 1.

**Table 1:** Baseline demographic and clinical characteristics of study participants (n = 124)

Parameter	Mean ± SD/n (%)
Age (years)	39.6 ± 12.8
Gender	
- Male	74 (59.7)
- Female	50 (40.3)
BMI (kg/m^2^)	25.3 ± 3.7
Indication for doxycycline	
- Respiratory tract infections	60 (48.4)
- Skin/soft tissue infections	34 (27.4)
- Pelvic inflammatory disease	30 (24.2)
Mean daily dose (mg)	200 ± 0
Duration of therapy (days)	10.7 ± 3.1

At baseline, all participants had normal serum pancreatic enzyme levels and no clinical or radiological evidence of pancreatitis. During the study period, seven patients (5.6%) developed acute pancreatitis confirmed by both biochemical and radiological findings as per the revised Atlanta criteria. The mean time to onset of symptoms after initiation of doxycycline therapy was 8.1 ± 2.3 days. The incidence of acute pancreatitis was slightly higher among females (3/50; 6.0%) compared to males (4/74; 5.4%), though the difference was not statistically significant (p = 0.84). The mean age of patients who developed pancreatitis was 42.7 ± 11.3 years, compared to 39.4 ± 12.9 years in those who did not (p = 0.41). Similarly, no significant association was observed between BMI and pancreatitis (p = 0.56). However, the duration of doxycycline therapy was significantly longer in those who developed pancreatitis (12.3 ± 2.1 days) compared to those who did not (10.6 ± 3.1 days; p = 0.03). The relationship between patient characteristics and pancreatitis occurrence is shown in Table 2.

**Table 2:** Association between patient characteristics and occurrence of doxycycline-induced acute pancreatitis (n = 124) * Independent sample t-test; ^†^ Chi-square test.

Variable	Developed Pancreatitis (n = 7; 5.6%)	Did Not Develop (n = 117; 94.4%)	P-value
Age (years), mean ± SD	42.7 ± 11.3	39.4 ± 12.9	0.41*
Gender, n (%)			
- Male	4 (5.4)	70 (94.6)	0.84^†^
- Female	3 (6.0)	47 (94.0)	-
BMI (kg/m^2^), mean ± SD	26.1 ± 3.2	25.2 ± 3.8	0.56*
Duration of therapy (days), mean ± SD	12.3 ± 2.1	10.6 ± 3.1	0.03*
Dose (mg/day), mean ± SD	200 ± 0	200 ± 0	-
Indication (respiratory vs. others), n (%)	4 (6.7)	56 (93.3)	0.72^†^

Among the seven patients with confirmed acute pancreatitis, all (7/7; 100%) presented with severe epigastric pain radiating to the back, accompanied by nausea and vomiting. Laboratory findings showed markedly elevated serum pancreatic enzymes, with mean amylase 422 ± 118 U/L and mean lipase 698 ± 210 U/L (normal reference <100 U/L). Liver and renal function parameters remained within normal limits in all patients. A comparison of baseline and post-therapy laboratory values for all participants is presented in Table 3.

**Table 3:** Comparison of baseline and post-therapy laboratory parameters in study participants (n = 124) A paired t-test was applied. Data are presented as mean ± SD. * means significant values at p < 0.05. ALT: alanine aminotransferase; AST: aspartate aminotransferase; ALP: alkaline phosphatase

Parameter	Baseline (Mean ± SD)	End of Therapy (Mean ± SD)	P-value
Serum amylase (U/L)	64.5 ± 18.2	82.1 ± 115.3	<0.001*
Serum lipase (U/L)	71.3 ± 24.6	97.4 ± 165.1	<0.001*
ALT (U/L)	29.7 ± 8.1	30.5 ± 9.4	0.48*
AST (U/L)	27.4 ± 7.6	28.1 ± 8.2	0.61*
ALP (U/L)	85.2 ± 25.3	86.7 ± 27.1	0.74*
Total bilirubin (mg/dL)	0.83 ± 0.21	0.87 ± 0.26	0.43*
Urea (mg/dL)	25.4 ± 7.9	26.2 ± 8.1	0.59*
Creatinine (mg/dL)	0.96 ± 0.21	0.98 ± 0.25	0.66*
Triglycerides (mg/dL)	145.6 ± 33.7	148.3 ± 36.1	0.57*

**Figure 1:**
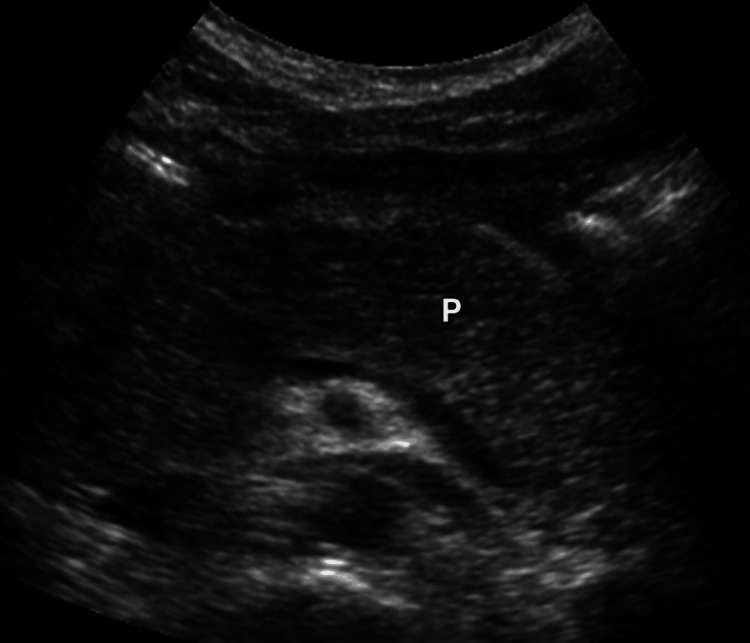
Ultrasonographic appearance of acute pancreatitis in symptomatic patients

Abdominal ultrasonography was performed in all symptomatic patients (Figure 1). It was diagnostic in five (71.4%) cases, while two (28.6%) required confirmation with contrast-enhanced CT scanning, which revealed diffuse pancreatic enlargement and peripancreatic fat stranding without necrosis. Based on the modified Glasgow-Imrie score, six patients (85.7%) had mild pancreatitis and one patient (14.3%) had moderate disease. None developed necrosis, pseudocyst, or multiorgan failure. The mean duration of hospital stay among patients with pancreatitis was 7.4 ± 2.2 days, and all (7/7; 100%) recovered fully with conservative management. No mortality occurred during the study. Details of clinical course and outcomes among affected patients are summarized in Table 4.

**Table 4:** Clinical course and outcomes among patients with doxycycline-induced acute pancreatitis (n = 7; 5.6%)

Parameter	Mean ± SD/n (%)
Time to onset of symptoms (days)	8.1 ± 2.3
Amylase (U/L)	422 ± 118
Lipase (U/L)	698 ± 210
Imaging findings	
- Ultrasonography positive	5 (71.4)
- CT-confirmed cases	2 (28.6)
Severity (modified Glasgow-Imrie)	
- Mild	6 (85.7)
- Moderate	1 (14.3)
Hospital stay (days)	7.4 ± 2.2
Complications	None
Mortality	None

## Discussion

In this prospective study, we observed an overall incidence of DIAP of 5.6% (7/124) among patients receiving doxycycline therapy. This incidence, although higher than earlier estimates, highlights the clinical importance of systematic monitoring for this rare adverse effect. DIP is estimated to account for only 0.1-2% of all acute pancreatitis cases worldwide, and tetracyclines, including doxycycline, are recognized as probable causative agents within this spectrum [1]. Variability in reported incidence rates likely reflects differences in study design, case identification, and diagnostic criteria. Our higher rate may be attributed to active, prospective surveillance and serial biochemical assessments, in contrast to passive case detection used in retrospective studies and case reports [2,4].

The mean age of our participants (39.6 ± 12.8 years) and a slight male predominance (59.7%) contrast with earlier case series that reported a female preponderance among published DIAP cases [6,7]. This discrepancy could reflect reporting bias in the case literature, where unusual or severe female cases are more likely to be published, rather than a true sex-linked predisposition. Similar to previous reports, no statistically significant association was found between age, BMI, or sex and the occurrence of pancreatitis [12]. Doxycycline was most often prescribed for respiratory tract infections, followed by skin and pelvic infections, paralleling prior prescribing trends reported in published literature [13].

The mean latency period from drug initiation to symptom onset in our study was 8.1 ± 2.3 days, consistent with previously documented ranges of 1-22 days and mean latencies of approximately 7-10 days [4]. A significant relationship between longer treatment duration and pancreatitis occurrence was found in our cohort (p = 0.03), suggesting a possible cumulative or time-dependent mechanism. While previous case reports have described both early-onset and delayed presentations, most cases occur within the first two weeks of therapy [6]. The uniform dosing regimen (200 mg/day) and mild disease severity in our cases align with prior findings indicating that DIAP is generally independent of dose intensity [14].

All affected patients presented with classic epigastric pain radiating to the back, accompanied by nausea and vomiting, symptoms indistinguishable from acute pancreatitis due to other causes [4,15]. Laboratory results showed substantial elevations of serum amylase (422 ± 118 U/L) and lipase (698 ± 210 U/L), satisfying the revised Atlanta criteria for diagnosis [16]. Similar enzyme profiles have been consistently reported in prior doxycycline-induced cases [17,18]. Liver and renal functions remained within normal limits in our patients, indicating that organ impairment secondary to systemic toxicity was unlikely, a finding consistent with other reports describing DIAP as an isolated pancreatic reaction [7].

Ultrasonography confirmed pancreatitis in five patients (71.4%), while two (28.6%) required confirmation with contrast-enhanced CT scans, which revealed diffuse pancreatic swelling and peripancreatic fat stranding without necrosis. This pattern corresponds to the mild-moderate disease spectrum typically observed in DIP [4]. Based on the modified Glasgow-Imrie criteria, six patients (85.7%) had mild, and one (14.3%) had moderate pancreatitis. These proportions are comparable to earlier case reports where the majority of DIAP cases were self-limited and resolved with supportive management [4-8]. All our patients recovered completely, and no mortality occurred, mirroring the favorable outcomes seen in most previous reports [4-8].

The precise mechanism of doxycycline-induced pancreatitis remains uncertain. Postulated mechanisms include direct pancreatic toxicity, toxic metabolite accumulation, hypersensitivity reactions, and increased biliary concentrations of tetracyclines leading to localized pancreatic injury [16]. The latency period of approximately one week, the absence of a dose-response relationship, and the predominance of mild cases in our study favor an idiosyncratic or hypersensitivity-mediated mechanism rather than direct cytotoxicity [15]. The higher biliary excretion of tetracyclines, including doxycycline, compared to serum levels may promote local pancreatic exposure and inflammation, a mechanism supported by pharmacokinetic data from related tetracyclines [4].

Rechallenge testing, though considered the gold standard for confirming drug causality, is ethically unjustifiable in suspected DIP. Consequently, most published cases and systematic reviews categorize doxycycline as a probable rather than definite cause of pancreatitis using the World Health Organization-Uppsala Monitoring Centre (WHO-UMC) and Naranjo criteria [4,7]. Our study employed strict exclusion criteria, removing confounding etiologies such as gallstones, hypertriglyceridemia, alcohol use, or concurrent pancreatotoxic medications, thereby enhancing the likelihood of true drug attribution despite the absence of rechallenge confirmation.

The present study expands on earlier case-based literature by providing the first prospective dataset assessing incidence and clinical characteristics of DIAP. Our observed latency (8.1 days) and predominance of mild disease are in agreement with most reported case studies [4-8]. However, our observed incidence (5.6%) is higher than the 0.1-2% reported in prior retrospective reviews [4,16,19-21]. This discrepancy likely results from our active follow-up strategy, which allowed early enzyme testing and prompt identification of mild or subclinical cases that might otherwise go unreported. Prior case reports, being anecdotal, could not determine incidence or identify potential predictors, underscoring the value of our systematic approach in quantifying risk.

The strengths of this study include its prospective design, structured data collection, baseline enzyme screening, application of the revised Atlanta diagnostic criteria, and standardized follow-up. Together, these minimize misclassification bias and strengthen causal inference. Nevertheless, certain limitations should be acknowledged. The number of pancreatitis cases was small, limiting multivariate analysis. The single-center design may restrict generalizability. Although we excluded common etiologies, subclinical biliary pathology could not be entirely ruled out. Finally, lack of rechallenge prevents confirmation of definitive causality, a limitation shared by virtually all prior DIAP studies.

Given doxycycline’s widespread use in clinical practice, awareness of this potential adverse effect is essential. The temporal clustering of cases within the first two weeks of therapy suggests that clinicians should maintain a high index of suspicion for DIAP in patients who develop unexplained abdominal pain during doxycycline treatment. Early discontinuation of the drug and supportive therapy usually results in complete recovery, as evidenced in our cohort and supported by prior literature [16,20,21]. Routine enzyme monitoring during prolonged doxycycline therapy may aid early detection in at-risk individuals.

To elucidate the true epidemiology and pathogenesis of DIAP, larger multicenter prospective studies are warranted. Population-based pharmacoepidemiologic investigations linking antibiotic prescriptions to hospitalization and laboratory databases could establish absolute risk estimates. Mechanistic studies focusing on genetic susceptibility, immunologic pathways, and metabolite analysis may clarify the biological underpinnings of this idiosyncratic reaction.

## Conclusions

Our findings confirm that doxycycline, although generally safe, can precipitate acute pancreatitis in a small subset of patients, particularly following therapy extending beyond 10 days. The clinical presentation is indistinguishable from pancreatitis of other etiologies, but is typically mild and resolves with drug discontinuation. These results substantiate prior case reports and provide the first prospective data quantifying risk and timing, thereby underscoring the importance of clinical vigilance and judicious use of doxycycline.
